# Nanoparticle drug delivery systems for synergistic delivery of tumor therapy

**DOI:** 10.3389/fphar.2023.1111991

**Published:** 2023-02-16

**Authors:** Daoyuan Chen, Xuecun Liu, Xiaoyan Lu, Jingwei Tian

**Affiliations:** ^1^ School of Pharmacy, Key Laboratory of Molecular Pharmacology and Drug Evaluation (Yantai University), Ministry of Education, Collaborative Innovation Center of Advanced Drug Delivery System and Biotech Drugs in Universities of Shandong, Yantai University, Yantai, China; ^2^ Shandong Boan Biotechnology Co., Ltd., Yantai, China

**Keywords:** nanoparticles, co-delivery, synergistic action, tumor therapy, combination therapy

## Abstract

Nanoparticle drug delivery systems have proved anti-tumor effects; however, they are not widely used in tumor therapy due to insufficient ability to target specific sites, multidrug resistance to anti-tumor drugs, and the high toxicity of the drugs. With the development of RNAi technology, nucleic acids have been delivered to target sites to replace or correct defective genes or knock down specific genes. Also, synergistic therapeutic effects can be achieved for combined drug delivery, which is more effective for overcoming multidrug resistance of cancer cells. These combination therapies achieve better therapeutic effects than delivering nucleic acids or chemotherapeutic drugs alone, so the scope of combined drug delivery has also been expanded to three aspects: drug-drug, drug-gene, and gene-gene. This review summarizes the recent advances of nanocarriers to co-delivery agents, including i) the characterization and preparation of nanocarriers, such as lipid-based nanocarriers, polymer nanocarriers, and inorganic delivery carriers; ii) the advantages and disadvantages of synergistic delivery approaches; iii) the effectual delivery cases that are applied in the synergistic delivery systems; and iv) future perspectives in the design of nanoparticle drug delivery systems to co-deliver therapeutic agents.

## 1 Introduction

Cancer is the second leading cause of mortality, followed by cardiovascular disease (Siegel et al., 2022). With the advancement of medical care in recent years and improved living standards, cancer mortality rate has decreased steadily (Torre et al., 2016). However, the number of diagnosed cancers has declined slightly since 2020, but the deaths have gradually increased because of untimely diagnosis and treatment, multiple viral infections, and slow information collection due to the COVID-19 pandemic (Yabroff et al., 2022). There were 1.91 million new cancer patients in the United States in 2022, of which 600,000 died. Prostate cancer was the most common cancer in men, breast cancer was the most diagnosed in women, and lung cancer, the primary cause of cancer death, resulted in approximately 350 deaths per day (Siegel et al., 2022). In clinical oncology, despite developing new cancer treatment approaches, such as the application of immunotherapy, RNAi therapy, and gene editing, chemotherapy (cytotoxic drugs) is still the principal therapeutic technique (Xin et al., 2017; Gupta et al., 2019). Although it plays an important role in treatment, it is associated with multidrug resistance (MDR), toxicity, and several side effects resulting in low patient compliance (Sun et al., 2014).

MDR is a major challenge in cancer therapy and leads to low intracellular drug concentrations and severe systemic toxicity, which could lead to chemotherapy failure (Bukowski et al., 2020). MDR results from the interaction of multiple mechanisms, and an accurate understanding of these mechanisms is helpful in cancer treatment (Jabr-Milane et al., 2008). Overexpression of membrane transporter proteins is a major drug resistance mechanism, which increases the efflux of substances from cells (Wei et al., 2019). In addition, reducing drug uptake and eliminating receptors and transporter proteins on the tumor surface results in insufficient drug uptake, affecting drug concentrations (International Transporter et al., 2010). Furthermore, DNA repair, anti-apoptotic and pro-apoptotic proteins, the complex tumor microenvironment (TME), and autophagy also promote the development of tumor MDR (Hu et al., 2012; Ostman, 2012; Wilson et al., 2012; Yang et al., 2018; Assaraf et al., 2019; Tarasov et al., 2019). Fortunately, nanomedicine shows great potential to overcome the variable MDR mechanisms which limit conventional chemotherapeutics.

Nano drug delivery systems (NDDS) provide a promising approach to controlled and targeted drug delivery and are one of the prospective strategies in cancer therapy (Chidambaram et al., 2011). Generally, nanoparticles can be prepared using organic and inorganic materials, such as lipids, polymers, and gold (Shi et al., 2017; Ulldemolins et al., 2021). As the particle size is approximately 100 nm, nanoparticles can utilize the enhanced permeability and retention effect (EPR) to passively target the tumor and remain in it (Danhier, 2016). Moreover, the physicochemical properties of the nanoparticles, such as size, structure, and surface charge, can be adjusted by the material composition and proportion (Lobatto et al., 2011; Wang et al., 2012). These biological properties give nanoparticles several advantages, such as tumor-targeted delivery, decreased systemic side effects, prolonged plasma circulation, *etc* (Markman et al., 2013; Wei et al., 2021). Nanoparticle drug delivery systems can also carry larger drug payloads and prevent recognition by efflux pumps (Scarano et al., 2015).

Owing to the complex tumor environment, effective treatment may not be achieved by single chemotherapeutic drugs or sequence-specific nucleic acids, thereby motivating the co-delivery of multiple therapeutic agents (Jang et al., 2016). Furthermore, combination delivery also has synergistic effects of elevating tumor inhibition efficiency through several distinct targets, and it is possible to reduce side effects and maximize drug efficacy (Eftekhari et al., 2019). Moreover, different types of cancers can be treated by co-administration therapy (Taratula et al., 2011; Yin T. et al., 2015). One of the most common strategies to increase the sensitivity of tumors to therapeutic agents is using nanoparticles to deliver two or more cytotoxic drugs (Hajipour et al., 2019). Therefore, combining cytotoxic drugs and nucleic acids may be another antitumor mode that can reduce drug dosage and reverse drug resistance (Chitkara et al., 2016). Recently, the co-delivery of genes and gene agents is an emerging pattern achieving synergistic regulation of gene expression in tumor cells (Pho-Iam et al., 2021).

**FIGURE 1:**
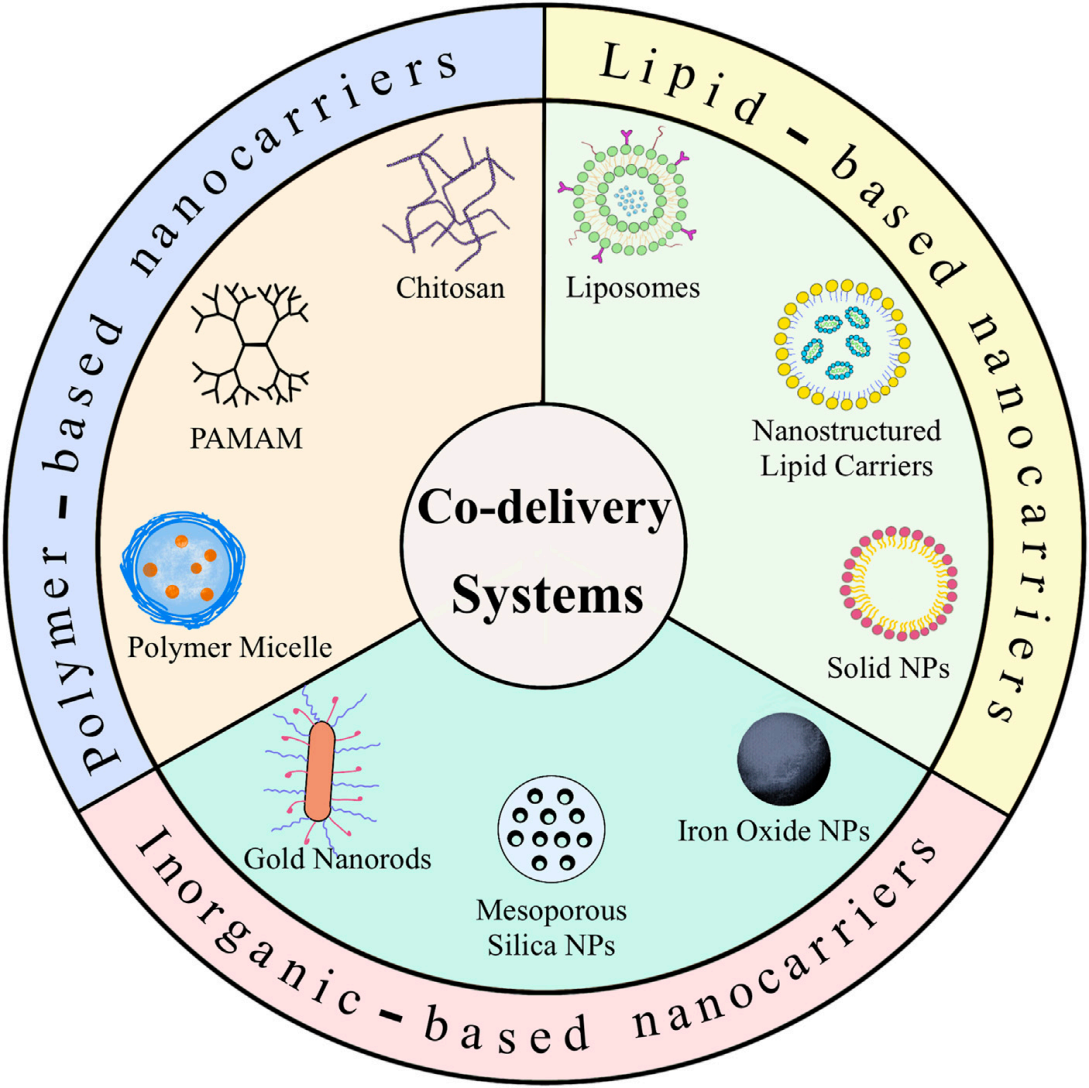
Schematic illustration of different nanocarriers for co-delivery of anticancer agents.

Alternative materials such as lipids, polymers and inorganic nano-systems have been used to fabricate these co-delivery nanoparticles. This review introduces the recent advances in co-delivery nanocarriers for cancer treatment, as shown in Figure 1, including i) the basic delivery strategy responding to several specific situations; ii) the preparation of three different materials for nanocarriers and successful applications of co-delivery; iii) a discussion of the value of this new approach, the future prospects and technical challenges in this field.

## 2 Lipid-based nanocarrier for co-delivery

**FIGURE 2:**
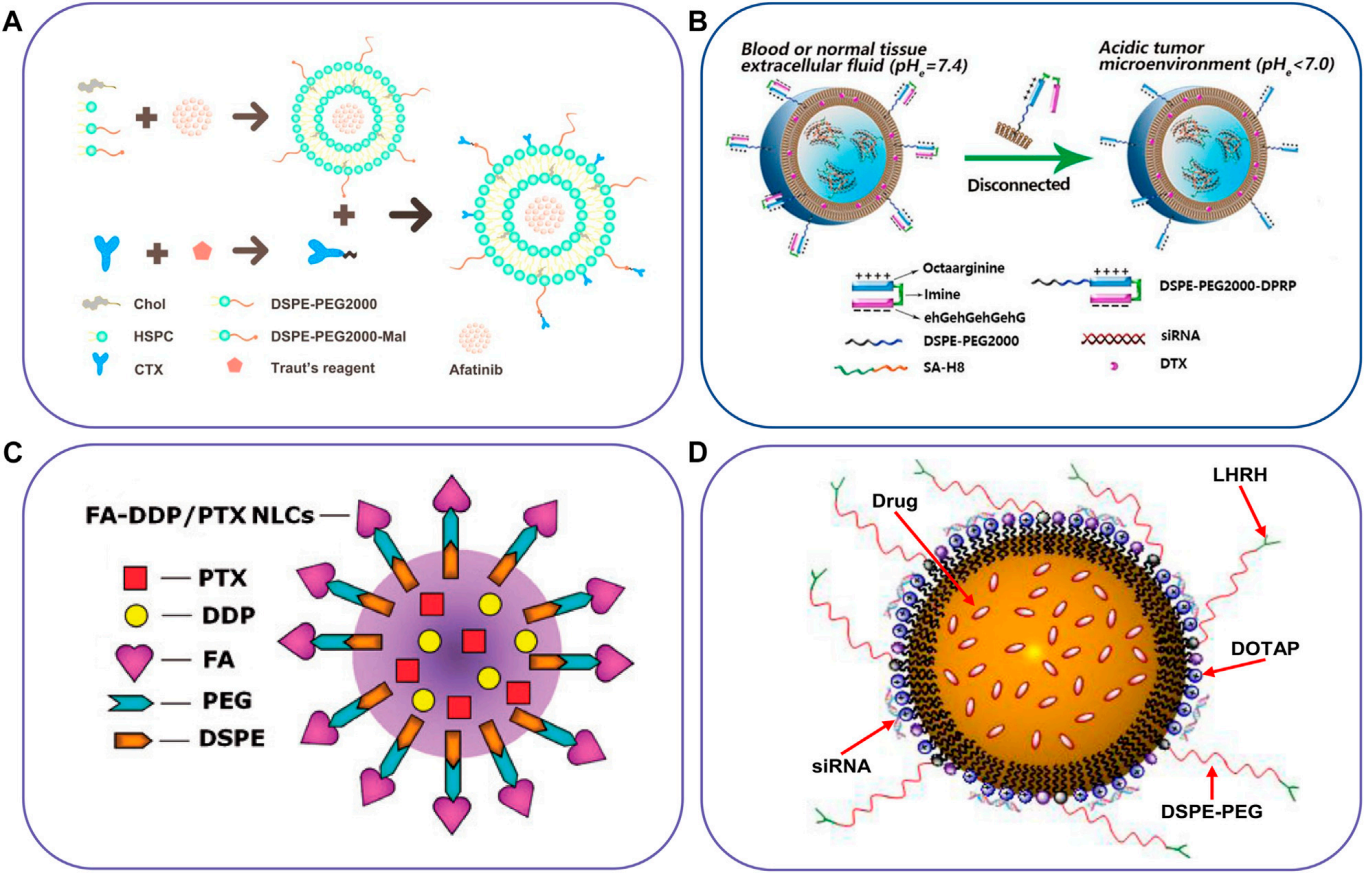
Schematic illustration of lipid-based nanocarriers to co-deliver two different agents. **(A)** Immunoliposome to deliver AFT and CTX, **(B)** pH-responsive liposome co-delivery DTX and siRNA, **(C)** FA-targeted NLC for co-delivery of DDP and PTX, and **(D)** NLC for co-delivery of an anticancer drug, siRNA and targeting peptide. Reproduced with permission from ref. (Lu et al., 2019), (Zhao et al., 2022), (Yang et al., 2016), (Taratula et al., 2013). Copyright 2019, 2022, 2016 and 2013, Elsevier, BioMed Central, Taylor & Francis, and Elsevier, respectively.

Lipid-based nanoparticles have many advantages in drug delivery systems, such as *in vivo* stability, high drug loading efficiency, biocompatibility, avoiding the use of organic solvents, and controllable drug release modes in the preparation (Sheoran et al., 2022). They can efficiently deliver nucleic acid and cytotoxic drugs (Aghamiri et al., 2019; Hajipour et al., 2019) and have been applied in various fields, such as biopharmaceuticals and food safety (Ghanbarzadeh et al., 2016). Due to the different manufacturing processes and lipid compositions, lipid-based nanoparticles have different physical and chemical properties and spatial structures, which also lead to different types of lipid nanoparticles, including liposomes, micelles, nanoemulsions, nanostructured lipid carriers, vesicles, and solid lipid nanoparticles (Tran et al., 2017). Several successful examples about schematic illustration of lipid-based nanocarriers to co-delivery agents have shown in Figure 2 and some innovative cases of lipid-based nanocarriers for simultaneous delivery have shown in Table 1.

**TABLE 1:** Lipid-based nanocarriers for simultaneous delivery of therapeutics for cancer treatment.

Nanocarrier type	Nanocarrier composition	Therapeutics	Cell lines	Indication	Refs
Liposome	Tf-PEG_3400_-DOPE, ePC, CHEMS	Cobimetinib/Ncl-240	HCT 116	Colon cancer	Sriraman et al. (2015)
Liposome	Maleimide-PEG_5k_, DOPE, EPC	Paclitaxel/Trichosanthin	A549	Lung cancer	Chen et al. (2017d)
Liposome	Cholesterol, PC, Film hydration/Cardiolipin	6-mercaptopurine/Daunorubicin	Jurkat/Hut78	Acute myeloid leukemia	Agrawal et al. (2005)
SLN	DSPE-PEG_3400_-Mal, SBL, DCC	Cisplatin prodrug/Paclitaxel	HeLa	Cervical cancer	Liu et al. (2017)
Liposome	DSPE-PEG_2000_, DPPC, DDAB	Docetaxel/siRNA	A549/H226	Lung cancer	Qu et al. (2014)
SLN	HA-as-DSPE, GM, SPC	Paclitaxel/pDNA	MCF-7	Breast cancer	Yu et al. (2016)
Liposome	EPC, DSPE-PEG_2000_, GE11	Gemcitabine/siRNA	Panc-1	Pancreatic cancer	Lin et al. (2019)
cSLN	DPhPE, DC-Chol, mPEG-DSPE	Paclitaxel/siRNA	KB	Oral epidermal cancer	Yu et al. (2012)
Liposome	DOTAP, DOPE, DSPE-PEG	Mitomycin C/siRNA	TR4	Bladder cancer	Cui et al. (2015)
SLN	DOTAP, DDAB, DOPE	pDNA/siRNA	HepG2	Liver cancer	Zhu et al. (2022)

### 2.1 Lipid-based nanocarriers for delivery of drugs and drugs

In 1965, it was first reported worldwide that combined regimens of chemotherapy drugs could be used to treat acute lymphoblastic leukemia. Researchers compared the use of methotrexate (MTX) plus 6-mercaptopurine (6-MP) versus single-drug therapy and found that the dual-drug regimen demonstrated great synergistic effect and had better efficacy in reducing the tumor size and alleviating the disease burden in children with acute lymphoblastic leukemia (Frei et al., 1965). However, the limitations of this regimen were low bioavailability, poor biocompatibility and drug leakage (Garcia-Pinel et al., 2019). Encouragingly, since then, there has been significant progress achieved in the study of delivery carriers and lipid-based co-delivery nanocarriers (Zununi Vahed et al., 2017).

#### 2.1.1 Liposome

Liposomes are spherical and have a phospholipid bilayer composed of phospholipid and cholesterol, which can simultaneously encapsulate hydrophilic and hydrophobic molecules (Lu et al., 2019; Shah et al., 2020), making them an excellent delivery carrier for drug co-delivery. Doxorubicin (DOX) and paclitaxel (PTX) are highly active anti-cancer drugs often used to treat non-small cell lung cancer, breast cancer, etc. (Wu et al., 2017), but their clinical use might be limited due to significant adverse events such as low solubility and multi-drug resistance. Franco et al. (Franco et al., 2019) used long-circulating and fusogenic liposomes (LCFL) constructed using lipid film hydration to deliver PTX and DOX to treat breast cancer. They found that the neutralized Zeta potential mean value of LCFL-PTX/DXR contained PEG on its bilayer. Further experiments using MTT and migration assays showed that co-delivery treatments were associated with lower IC_50_ values and reduced percentage of cell migration than treatment with free PTX or DOX. In addition, they observed that compared with free PTX or DOX, the long circulation fusion liposome encapsulated with PTX:DOX 1:10 M ratio had better anti-tumor effects and significantly improved cardiotoxicity; thus, they concluded that combined administration of LCFL PTX/DXR could achieve more positive results compared with PTX and DOX alone for the treatment of breast cancer.

In an innovative study by Li et al. (Li M. et al., 2022), the investigators co-delivered crizotinib (Cri) and F7 into thermosensitive liposomes (TSL) for treating breast cancer. F7 is a new drug that can significantly inhibit cell proliferation, but its application is often limited due to its high toxicity and drug resistance with Cri. The F7-Cri-TSL regimen demonstrated good stability, heat sensitivity, and synergistic therapeutic effects with reduced systemic cytotoxicity. Therefore, F7-Cri-TSL was proposed as a thermosensitive treatment for breast cancer. Gao et al. (Gao et al., 2022) used microfluidic technology to design a co-drug delivery system using curcumin (CUR) and prodrug SN38 for treating lung cancer. They linked SN38 to the cell-penetrating peptide TAT through the polyethylene glycol (PEG) linker, which was then co-loaded with liposomes and CUR to form the liposome-TAT-PEG-SN38/CUR complex (size, 171.21 nm). Their results showed that Lip TAT-PEG-SN38/CUR could significantly inhibit cell proliferation, increase cell apoptosis and demonstrated significant anti-tumor effects.

#### 2.1.2 Nanostructured lipid carriers

Nanostructured lipid carriers (NLC) are second-generation drug carriers based on lipid nanoparticles that can enhance the stability of carriers and accurately control drug release (Maroufi et al., 2020). NLC has a large internal regulation space conducive to delivering hydrophobic drugs (Doktorovova et al., 2014; Weber et al., 2014). Because of the different mechanisms of action of PTX and DOX, the combination delivery demonstrated favorable results in treating solid tumors. Wang et al. (Wang et al., 2016) used melt emulsification technology to prepare PTX and DOX nano lipid carriers to study cytotoxicity in non-small cell lung cancer cell lines. Their viability results using NCL-H460 cells showed that the cytotoxicity effects of PTX-DOX-NLC on lung cancer cells were 3 times higher than that of single drug NLC and 9-fold better than the free drug formula. Compared with single-drug NLC, PTX-DOX-NLC had higher tumor targeting and stronger anti-tumor activity, reduced systemic toxicity and greater efficacy in inhibiting lung cancer growth, suggesting an effective strategy for the targeted treatment of lung cancer.

Yang et al. (Yang et al., 2016) jointly delivered cisplatin (DDP) and paclitaxel (PTX) to treat head and neck cancer by constructing folic acid-modified nano lipid carriers (NLCs). In previous studies, although DDP plus PTX had more distinctly auxiliary value than single drug therapy, the adverse events should be ignored. The results showed that FA-DDP/PTX-NLC had significant inhibitory and synergistic effects on head and neck cancer cells (FaDu cells) and improved the anti-cancer efficiency of tumor-bearing mice. Therefore, the NLC-based FA-DDP/PTX carrier increased drug loading and sustained release, demonstrating promising potential as a targeted molecular drug for treating head and neck cancer. To overcome the MDR of combination chemotherapy, Jiang et al. (Jiang et al., 2016) developed an SLN by solvent injection technique to co-load CUR and etoposide (ETP), an inhibitor of DNA topoisomerase II that can affect the G2 phase of the cell cycle. *In vitro* cell viability studies using SGC 7901 cell lines showed that the cytotoxicity values of ETP-CUR-NLCs were significantly higher than that of drug solution samples. The tumor tissue distribution of ETP in ETP-CUR-NLCs was significantly higher than in other tissues and demonstrated stable blood-drug concentration during the tumor therapy.

#### 2.1.3 Other lipid-based carriers

In general, the entrapment efficiency and drug loading of liposomes would be affected by the complex environment, resulting in drug leakage and decreased efficacy (Large et al., 2021). The use of nanoemulsion nanocarriers may help to solve this problem. Due to their high specific surface area, tissue targeting and long circulation characteristics, nanoemulsions have started to be used in the clinical treatment of malignant tumors (Sanchez-Lopez et al., 2019). To solve the problems of low solubility and bioavailability of bicalutamide (BCT), Arya et al. (Arya et al., 2017) used a nanoemulsion drug delivery system to co-deliver BCT and hesperidin (HSP) to treat prostate cancer. The results showed that the nanoparticles had smaller particle sizes and faster drug release. The entrapment rates of BCT and HSP were 91.29% and 88.19%, respectively. Moreover, the significant decrease in the level of biochemical markers of nephrotoxicity showed that the tissue toxicity was significantly reduced and that BCT treatment could alleviate pulmonary fibrosis.

Nano-miceller drug delivery carriers could be used to improve the internal absorption efficiency of drugs such as the co-delivery tamoxifen (TMX) and naringenin (NG) for the treatment of breast cancer (Sandhu et al., 2017). Investigations on the uptake of Caco-2 cells showed that the cell uptake potential of TMX-NG – SNEDDS was high and that the cytotoxicity of TMX-NG-SNEDDS to MCF-7 cells was significant. The drug release curve showed that TMX-NG-SNEDDS, prepared using natural lipids and biocompatible excipients, could completely release the drug within 30 min, with better biocompatibility and anti-cancer effects. The phospholipid bilayer is the basic support of the cell membranes and also a natural drug delivery carrier (Liu et al., 2022). Researchers extracted the vesicles secreted by cancer cells to prepare drug delivery carriers, which effectively delivered drugs to cancer cells *via* direct fusion with cancer cells. Li et al. (Li H. et al., 2022) used extracellular vesicles (EVs) as natural drug carriers to co-deliver DOX and ronidamine (LND), a chemical sensitizer, for the treatment of lung cancer. They found that different centrifugal forces could affect the size of EVs, thus, affecting anti-cancer efficiency, and their results showed that smaller EVs had higher anti-cancer efficiency. The two drugs inhibited the proliferation of cancer cells *via* DNA damage, ATP inhibition and increased ROS production (Swift et al., 2006; Juan et al., 2008).

### 2.2 Lipid-based nanocarriers for delivery of drugs and genes

Combining two therapeutic agents with different mechanisms is becoming a promising strategy in cancer therapy (Fisusi and Akala, 2019). Integration between chemotherapeutics and genes may enhance the synergistic action and increase the efficiency of overcoming MDR (Qi et al., 2017). Two approaches are used in gene therapy, DNA therapy and RNAi therapy (Athanasopoulos et al., 2017). A plasmid DNA (pDNA) usually has high molecular weight and is a circular double-stranded DNA. It can be moderated to specific RNA-related genes and translated into proteins in the nucleus (Cheng et al., 2021). However, the difficult access to the nucleus blocks the function of pDNA and directly affects the intracellular level of gene expression. RNA interference therapy (RNAi) is an emerging therapeutic tool that can control gene expression in several diseases (Setten et al., 2019). Small interfering RNA (siRNA), short hairpin RNA (shRNA) and microRNA (miRNA) are the main members of RNAi (Weng et al., 2019). They can silence specific genes to restrict the overexpression of encoding proteins and accomplish the purpose of cancer treatment. The combination of drugs and genes may result in synergistic functions by inhibiting MDR, a major challenge of cancer therapies (Weng et al., 2019). Lipid-based nanocarriers were shown to be among the most successful approach for drug and gene delivery (Ansari et al., 2020). It can modify the physicochemical properties by connecting with different materials to improve co-delivery efficiency (Buya et al., 2021; Su et al., 2022).

#### 2.2.1 Liposome

Liposomes can be broadly used as delivery models because of their ability to carry nucleic acid agents and chemotherapeutic drugs and combination with lipids and components to encapsulate genes and drugs (Zhang Y. et al., 2021). The loaded agents can interact with lipid carriers and affect the co-delivery efficiency to tumors (Ickenstein and Garidel, 2019). Generally, it utilizes electrostatic interaction between cationic liposome and siRNA to form complexes. In addition, siRNA can be loaded in the core of liposomes when the carrier is similarly charged (Antipina and Gurtovenko, 2018; Song et al., 2021). To enhance the penetration into tumors, Zhao et al. (Zhao et al., 2022) designed a liposomal platform, D-L/si-DTX, to co-deliver DTX and PLK-1-siRNA using a pH-sensitive peptide, DPRP, for cancer therapy. The results showed that the advanced liposome complex, consisting of DSPE-PEG2000-DPRP, significantly promoted cellular uptake and adequate lysosome escape in the cytoplasm. Moreover, D-L/si-DTX exhibited tumor-selective delivery and inhibited tumor growth by improved penetration in tumor spheroids. *In vitro* and *in vivo* studies demonstrated that D-L/si-DTX could significantly downregulate the expression of PLK-1 and suppress tumor growth with accurate delivery and no adverse toxicity compared with single-loaded liposomes; indicating that their proposed delivery platform could become a promising strategy for combination therapy.

At present, gene delivery approaches are mainly based on siRNAs (Goncalves and Paiva, 2017). However, compared with siRNA, shRNA has the capability of multi-target silencing with superior efficiency, making shRNA a better therapeutic candidate (Moore et al., 2010). To improve cellular uptake, Swami et al. (Swami et al., 2021) developed a complex liposome system (DTX-lipoplex) to co-deliver DTX and SIRT1-shRNA for breast cancer treatment. The liposome was prepared by a solvent evaporation method, which was 200 nm in size. Their results showed that DTX-lipoplex had excellent stability in plasma circulation and could protect shRNA from degradation. Furthermore, passive targeting using nanoparticles could have higher efficiency in treating breast cancer. Chowdhury et al. (Chowdhury et al., 2017) was the first to co-deliver shPFKFB3 and docetaxel (DTX) using liposome to increase apoptosis and cellular stress for the treatment of NSCLC. They observed enhanced therapeutic efficacy of docetaxel with the RNAi-based glycolytic inhibitor and chemosensitization of NSCLC cells.

#### 2.2.2 Solid lipid nanoparticles (SLN) and nanostructured lipid carriers (NLC)

Solid lipid nanoparticles (SLN), as an alternative carrier of liposomes and polymer nanoparticles, have received extensive attention in the field of drug delivery due to their outstanding colloidal stability and low toxicity (Montasser et al., 2013). SLNs are nano-spherical particles prepared by dispersing solid lipids in an aqueous solution. Generally, SLN consists of a lipid core and amphiphilic surface (Ganesan et al., 2018). Owing to their non-water solid core and inner drug core, SLNs have a better protection capacity than liposomes (Liang et al., 2021). Nanostructured lipid carriers (NLC) are a new generation of lipid-based nanoparticles developed through different nanocarriers and can overcome the limitations of conventional lipid nanoparticles (Tapeinos et al., 2017). Therefore, NLCs have received considerable attention as an advanced drug delivery tool for cancer therapy.

Pemetrexed is a promising chemotherapeutic agent for the treatment of glioblastoma (Genova et al., 2013). To improve the treatment effects, Berrin et al. (Kucukturkmen and Bozkir, 2018) prepared cationic SLNs to co-deliver miR-21 and pemetrexed for glioblastoma treatment. The therapeutic agents were encapsulated by a high-pressure homogenization method. The cSLNs particle had a size of 125 nm and a zeta potential of 27 mV. The combined formulation was shown to prolong the circulation of pemetrexed and achieve diffusion-controlled release at body temperature. *In vitro* cellular properties tests showed that cSLNs significantly promoted U87MG cell uptake and demonstrated promising results in cytotoxicity-related experiments. An attractive strategy in drug delivery is the transportation of miRNAs and chemotherapeutics to their respective target sites. Shi et al. (Shi et al., 2014) developed a novel delivery system using SLNs for miR-34a-microRNA and paclitaxel (PTX) for melanoma therapy. The average particle size of this nanoparticle was 220 nm and demonstrated excellent protection from degradation in the serum. *In vivo* experiments showed that passive targeting was the main pattern in lung tissues and that PTX exhibited enhanced activity upon synergizing with miR-34a. This co-delivery system demonstrated promising potential as a new approach for glioblastoma therapy.

Due to overexpression in cancer cells, transferrin (Ff) has been widely used in drug delivery platforms as actively targeting ligands (Gomme et al., 2005). To achieve higher loading capacity, Shao et al. (Shao et al., 2015) designed transferrin (Tf)-decorated NLC for co-delivery with paclitaxel (PTX) and plasmid. The particle size of Tf-decorated NLC (Tf-PTX-DNA-NLC) with PEG_5k_ and PEG_10K_ were 135 nm and 235 nm, respectively. The delivery system exhibited low cytotoxicity and high gene transfection efficiency *in vivo*. The active targeting capacity to NCL-H460 cells was enhanced by Tf decoration. Traditional lung cancer treatment was achieved by intravenous chemotherapy and resulted in severe toxic effects on healthy tissues. Inhalation has been recently proposed as an ideal strategy for the optimal efficiency of anti-lung tumor therapy. To reduce the risk of adverse events, Taratula et al. (Taratula et al., 2013) synthesized a nanostructured lipid nanocarrier to co-delivery MRP1-siRNA and doxorubicin or paclitaxel for treating lung cancer by inhalation. Compared with intravenous treatment, the NLCs showed higher anti-tumor activity and lesser exposure to healthy tissues. Therefore, this delivery system might be a promising approach for treating lung carcinoma in the future.

#### 2.2.3 Other lipid-based carriers

The preparation of nanoparticles by microfluidic technology has led to a revolution in the field of drug delivery. Precise and controllable nanoparticle size represents a promising preparation scheme for the particles to effectively penetrate the tumor interstitial barrier. To improve the serum stability of nanoparticles, Younis et al. (Younis et al., 2021) developed usLNPs for the co-delivery of SOR and MK-siRNA to treat hepatic carcinoma. This usLNP had a size of 60 nm and exhibited tumor penetration performance. *In vivo* experiment results showed that the novel nanoparticles could eradicate HCC tumors at a low dose of therapeutic agents and demonstrated excellent biosafety, indicating a promising strategy for the treatment of SOR-resistant HCC. However, issues of large-scale production could be an obstacle in the future.

Recent studies showed that grafting sucrose laurate with liposome could enhance the efficiency of gene transfection (Samaridou et al., 2020). To improve the inhibition of cancer progression with synergistic efficacy, Zhang et al. (Zhang et al., 2020) prepared novel nanoparticles to co-encapsulate paclitaxel (PTX) and siRNA by using tripeptide lipid and folate-PEG_2000_-DSPE. In addition, sucrose laurate was used to achieve higher gene transfection efficiency to graft the surface of LNP. This advanced nanoparticle improved cell uptake and controlled release, which inhibited tumor growth by limiting VEFG expression. Moreover, a low dosage of PTX in the complex demonstrated similar effects as single-drug with high dosage, therefore showing good potential to become an effective delivery platform for cancer treatment.

Sorafenib (SRF) is a multikinase inhibitor and has both anti-tumor and anti-angiogenic effects, but its application is hindered by MDR (Kong et al., 2021). Wang et al. (Wang et al., 2019) developed antibody-targeted lipid nanoparticles (G-S27LN) to co-deliver SRF and miRNA against liver cancers. Their novel nanosystem released SRF in a pH-sensitive responsive manner, and their designed combination mode led to lower cell viability. Animal studies showed significant suppression of tumor growth with no toxicity with G-S27LN preparation. Therefore, this platform represents a promising strategy for breast cancer therapy.

### 2.3 Lipid-based nanocarriers for delivery of genes and genes

Gene therapy has also made significant progress with the development of delivery systems (Tang and Xu, 2020). Some specific diseases, such as liver cancer, need to upregulate oncogenes and downregulate tumor suppressor genes at the same time, so the exploitation of dual gene delivery system is also imminent (Xu et al., 2018). Due to different mechanisms of action in cells, delivery agents containing two genes may have more significant therapeutic effects than monogenic agents. Some delivery platforms for dual gene systems had been developed to treat gene dysregulations, such as siRNA/siRNA, miRNA/miRNA, pDNA/siRNA, *etc* (Li Y. et al., 2018; Sarker et al., 2019; Muhammad et al., 2020).

High efficiency of cell uptake and excellent lysosomal escape are the most outstanding advantages of cationic lipid nanoparticles, which have been widely used in siRNA delivery systems (Yonezawa et al., 2020). SiRNA is encapsulated in multilayer lipids and can be fully protected in systemic circulation. Therefore, Kim et al. (Kim et al., 2019) designed a ligand coupled cationic nanoparticles carrying siRNA and quantum dots (QD) for the treatment of breast cancer. Cationic lipids become coated with two siRNA therapeutics (Bcl-2 and PKC-l) and simultaneous QD incorporation. The results showed that QLs achieved enhanced targeting delivery to cancer cells and gene silencing efficiency. Moreover, the aptamo-QLs exhibited competitive therapeutic efficacy compared to immuno-QLs and may be a potential delivery carrier for RNA interference. To improve the bioavailability of gemcitabine, Simonenko et al. (Simonenko et al., 2020) co-delivered CHK1-siRNA and WEE1-siRNA by using lipid nanoparticles to treat pancreatic cancer. The results showed that co-delivering two siRNAs brought about a 10-fold efficacy improvement compared with gemcitabine alone. These systems may provide a novel thought for pancreatic cancer therapy. In addition, joint delivery of gene drugs also includes siRNA and mRNA, both types of RNA have made significant progress in recent years. In order to maximize the therapeutic effect, Ball et al. (Ball et al., 2018) explored the strategy of simultaneous delivery of siRNA and mRNA by single lipid nanoparticles. Due to the differences in the molecular weight and stability between siRNA and mRNA, specific reactions are required for co-delivery. Polyethylene glycol (PEG), cholesterol and two kinds of lipids (DSPC and DOPE) were selected as raw materials to prepare LNP, and the delivery effect was depended on adjusting the proportion of ingredients. The experimental results showed that LNP containing two kinds of RNA significantly enhanced the therapeutic effect *in vivo* and *in vitro*, doubled the gene silencing efficiency compared with siRNA alone (0.05 mg/mL), and tripled the luciferase expression compared with mRNA alone (0.5 mg/kg).

Chen et al. constructed liposome-polycation-hyaluronic acid nanoparticles using antibody fragment (scFv) to co-deliver siRNA and miRNA to treat melanoma lung metastasis (Chen et al., 2010). siRNA downregulated the target gene (c-Myc/MDM2/VEGF) in lung metastasis. In addition, miRNA-34a induced B16F10 cell apoptosis, inhibited survivin expression, and downregulated the MAPK pathway. The co-delivery of two kinds of RNA significantly reduced toxicity to normal tissues, enhanced the treatment, and demonstrated good clinical application prospects for cancer therapies as they promoted LPH nanoparticles to target scFv. In addition to siRNA, miRNA is also used in dual delivery. To improve cell uptake and plasma stability, Ghaffari et al. (Ghaffari et al., 2021) optimized cationic niosomes to target the Bcl-2 gene by co-encapsulating miR-15a and miR-16-1 for the treatment of prostate cancer. The particle size of the specific noisome was 70 nm and had a zeta potential of +14.83 mV. The combination therapy of nanocarriers significantly decreased expression of the Bcl-2 gene and promoted the cell death of PC3 cells, providing an applicable drug delivery approach against prostate cancer.

## 3 Polymer-based nanocarriers for co-delivery

**FIGURE 3:**
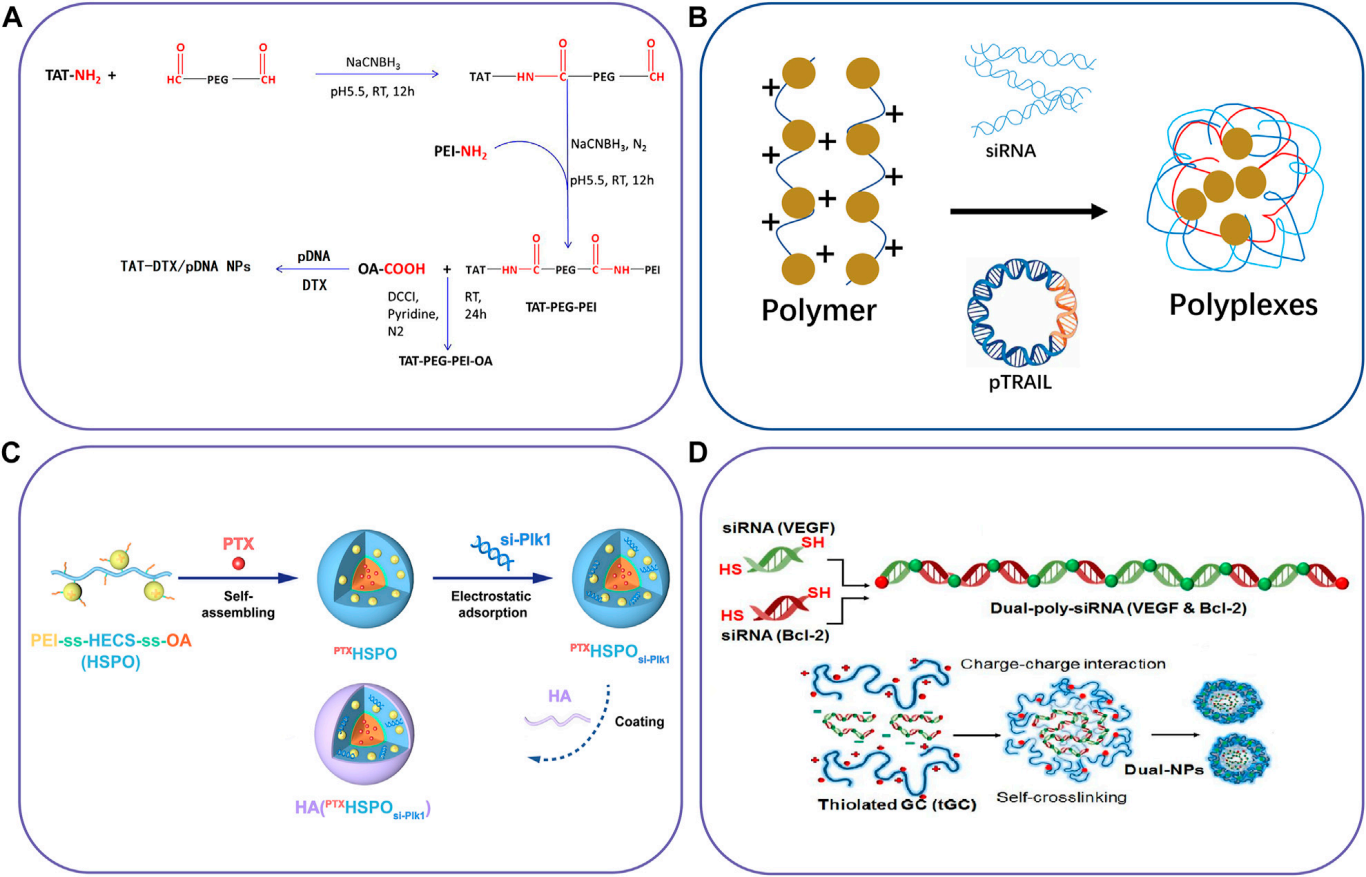
Schematic illustration of polymer-based nanocarriers to co-deliver two different agents. **(A)** The synthetic route of TAT-PEG-PEI-OA for co-deliver of DTX and pDNA, **(B)** lipopolymers to delivery pDNA and siRNA, **(C)** Chitosan derivatives for co-delivery of siRNA and PTX, and **(D)** Thiolated glycol chitosan for co-delivery of Dual-poly-siRNA. Reproduced with permission from ref. (Dong et al., 2016), (Thapa et al., 2019), (Yin et al., 2020), (Lee et al., 2015). Copyright 2016, 2019, 2020 and 2015, Elsevier, Mary Ann Liebert, American Chemical Society, and Elsevier, respectively.

The development of nanotechnology makes it possible for drugs to be absorbed or combined on the surface of nanoparticles, encapsulated in the core or dissolved in the particle matrix, and also makes it possible for targeted, safe and effective pharmaceutical preparations of nanoparticle (NPs) (Chaturvedi et al., 2019). Nanodrug delivery systems have shown great potential in improving the solubility of hydrophobic drugs, enhancing the drug’s biological distribution and pharmacokinetics, and providing preferential accumulation in targets (Zhang et al., 2019). Lipid-based nanoparticles have become the most common type of nano drugs approved by the FDA (Wang T. et al., 2021). However, the polymer-based nanocarrier is also considered an ideal drug delivery material because of its physical and chemical properties, such as biodegradability, biocompatibility, water solubility and storage stability (Ballarin-Gonzalez et al., 2014). Several instances about schematic illustration of polymer-based nanocarriers have shown in Figure 3 and some examples of polymer-based nanocarriers have shown in Table 2.

**TABLE 2:** Polymer-based nanocarriers for simultaneous delivery of therapeutics for cancer treatment.

Nanocarrier type	Nanocarrier composition	Therapeutics	Cell lines	Indication	Refs
Emulsion	PLGA, PVA, PHBV	5-Fluorouracil/oxaliplatin	HT-29	Colon cancer	Handali et al. (2019)
Emulsion	PLGA, Pluronic F127, chitosan, hyaluronic acid	Doxorubicin/irinotecan	MDA-MB-231/PC-3	Breast cancer/prostate cancer	Wang et al. (2015a)
Emulsion	PLGA, PVA, chitosan	Camptothecin/curcumin	CT26	Colon cancer	Xiao et al. (2015)
Emulsion	PLGA, PEG, FA	Paclitaxel/cisplatin	A549	Lung cancer	He et al. (2015)
Micelle	PEI, DA, Pullulan	Doxorubicin/pDNA	HL7702	Liver cancer	Chen et al. (2017c)
Micelle	DDAB, mPEG-PCL	Lycopene/siRNA	MCF-7	Breast cancer	Mennati et al. (2022)
Dendrimer	PAMAM, ERL, CQ	Erlotinib/pDNA	H1975	Lung cancer	Lv et al. (2018)
Emulsion	PLGA, PEI	Curcumin/siRNA	MCF-7	Breast cancer	Sarpoli et al. (2022)
Micelle	PEI, NCA, PSer	siRNA/pDNA	293T-GFP/HeLa	Cervical cancer	Chen et al. (2017b)
Peptide dendrimer	TFA, DIPEA, PyBop	siRNA/siRNA	A549	Lung cancer	Wu et al. (2021)

### 3.1 Synthetic polymer-based nanoparticles

Polymers can be divided into synthetic and natural polymers according to the synthesis method. Synthetic polymers include PLGA, PEI and PAMAM, while natural polymers include Poly (l-lysine) (PLL), chitosan (CH) and hyaluronic acid (HA) (Methachan and Thanapprapasr, 2017; Nayanathara et al., 2020; Gigmes and Trimaille, 2021). Various polymers have been studied to verify compliance with delivery systems’ requirements for improving delivery efficiency (Luque-Michel et al., 2017; Kang et al., 2021).

#### 3.1.1 PLGA

PLGA are synthetic polymers with properties of biodegradability and biocompatibility, approved by the FDA, and have shown potential as promising drug delivery materials (Sadat Tabatabaei Mirakabad et al., 2014). PLGA is hydrolyzed to lactic acid and glycolic acid monomers under acidic conditions (Ding and Zhu, 2018). The tricarboxylic acid cycle can metabolize these monomer units to avoid tissue toxicity caused by carrier accumulation (Kapoor et al., 2015). PLGA nanoparticles enter cells *via* endocytosis, and the retained PLGA nanoparticles slowly release the encapsulated drug resulting in sustained drug effects. To enhance the efficiency of PLGA nanoparticles entering lung cancer cells, Sharma et al. (Sharma et al., 2020) used polycationic polymer PEI to modify PLGA and co-deliver epirubicin (EPI) and paclitaxel (PTX) to detect the anti-cancer effects in A549 lung cancer cells. The particle size of PLGA-PEI-EPI-PTX was 241 nm, and the zeta potential was 42 mV. The research results showed that as the ester bond between lactic acid and glycolic acid monomer units broke, with the increase in solution acidity, the loading drugs continued to diffuse from the nanoparticles and maintained a controlled drug release. In addition, the synergistic effects of EPI with PTX in nanoparticles also effectively inhibited cell migration and invasion.

Stat3 are activator of transcription proteins of the Stat family that regulates apoptosis, cell cycle and angiogenesis. Overexpression of Star3 may lead to MDR, while inhibition of Star3 can reduce the activity of cancer cells and increase apoptosis (Zou et al., 2020). To improve the therapeutic effects of lung cancer, Su et al. (Su et al., 2012) used PEI on the surface of PLGA to co-deliver paclitaxel and siRNA (against Stat3). The nanocarrier was constructed in three steps. First, paclitaxel was encapsulated in PLGA nanoparticles by solvent evaporation. Then, PEI was coated on the surface of PLGA. Lastly, the final carrier was prepared by negatively adsorbing charged siRNA through electrostatic interaction. The particle size of PLGA-PEI-TAX-S3SI was 250 nm, measured by transmission electron microscopy. The complex was taken up by A549 and A549/T12 cells and demonstrated high cytotoxicity in these cells. The results of confocal microscopy showed that PLGA nanoparticles were still released 3 h after injection, indicating that the carrier had the ability to slowly release the drug. In addition, PLGA-PEI-TAX-S3SI was associated with more apoptosis than PLGA-PEI-TAX, demonstrating that it successfully inhibited the expression of Stat3.

Paclitaxel and cisplatin are widely used in the treatment of ovarian cancer, but due to MDR, their therapeutic effects in recurrent or advanced ovarian cancer remain limited (Wang H. et al., 2021). Studies have shown that drug efflux and the anti-apoptosis pathway could be the main factors for drug resistance in ovarian cancer (Khan et al., 2017). Therefore, Risnayanti et al. (Risnayanti et al., 2018) developed a “dual RNAi delivery system” to simultaneously deliver MDR1 and Bcl-2 siRNA to inhibit both MDR pathways. Their PLGA complex was synthesized using the double emulsion solvent evaporation method, and polylysine was used to reduce the negative electricity brought by siRNA. The particle size of the nanoparticles was 197 nm, and the Zeta potential was −2.5 mV. Compared with a single RNAi inhibition pathway, the dual RNAi delivery system broke the interdependence of the two mechanisms, significantly reduced the MDR of ovarian cancer cells, and significantly increased the toxicity of paclitaxel and cisplatin to cells. Thus, this system could be further evaluated as a promising treatment strategy for recurrent or advanced ovarian cancer.

#### 3.1.2 Polyethyleneimine (PEI)

Gene delivery vectors can be divided into viral vectors and non-viral vectors (Santana-Armas and Tros de Ilarduya, 2021). Viral vectors are rarely used due to significant safety concerns (Balakrishnan and David, 2019). PEI, as the representative of non-viral vectors, is widely used for gene delivery because of its outstanding biocompatibility and high transfection efficiency and is considered a gold standard of gene transfection (Lungwitz et al., 2005). Liver cancer immunotherapy has been the hotspot in liver cancer treatment. To induce anti-tumor immune responses, Pei et al. (Pei et al., 2022) designed nanovaccines consisting of polyanionic alginate (ALG) and polycationic polyethyleneimine (PEI) to co-deliver glypican-3 peptide antigens and unmethylated cytosine-phosphate-guanine adjuvants. The particle size of nanovaccines was 218 nm, and the zeta potential was 21 mV. Cellular uptake experiment showed that nanovaccines enhanced antigen and adjuvant uptake by dendritic cells and promoted endosomal escape of the peptide. In addition, the nanovaccines exhibited significant stimulation of DC maturation and also induced cytotoxic T lymphocyte responses. Therefore, this study could be a promising approach for liver cancer immunotherapy.

Docetaxel (DTX) is one of the most important chemotherapy drugs and the only drug for treating resistant prostate cancer (Assi et al., 2020). However, severe drug resistance hinders its clinical application. Because DTX has multiple drug resistance mechanisms, combining gene therapy with chemotherapy could be a promising durable strategy (Guo et al., 2019). Due to their different properties, using DNA and chemotherapy drugs in the same carrier delivery is challenging. TAT peptide is a transduction domain from HIV-1, which can improve the uptake of gene vectors and transfection efficiency. Dong et al. (Dong et al., 2016) synthesized an advanced PEI carrier that combined TAT peptide, oleic acid and PEG to form TAT-PEG-PEI-OA for delivering pDNA and DTX. The particle size of the complex was 270 nm, and the zeta potential was 22 mV. Cytotoxicity data showed that TAT-DTX/pDNA demonstrated significant tumor cell inhibition ability. In addition, after 24 h of administration, the transfection efficiency of TAT-DTX/pDNA was 1.5 times higher than the control group. These results indicated that the TAT-DTX/pDNA complex could allow continuous delivery of drugs in tumor tissues.

Tumor necrosis factor apoptosis-inducing ligand (TRAIL) can induce apoptosis in cancer cells while not affecting the survival of normal cells (Sloot et al., 2006). Researchers examined the use of TRAIL in treating p53 mutated cancer, but due to its short half-life and poor pharmacokinetic characteristics, the therapeutic effects of TRAIL remain unsatisfactory. Studies have shown that silencing Bcl-2 and SOD1 with siRNA could increase the sensitivity of TRAIL, and using pDNA could enhance the expression of required proteins (Thapa et al., 2018). Therefore, the simultaneous delivery of pTRAIL and Bcl-2 siRNA for breast cancer treatment is a promising approach. Thapa et al. (Thapa et al., 2019) grafted lipids onto PEI through thioester and amide linkage to prepare cationic polymers for the co-delivery of siRNA and pDNA to treat breast cancer. After delivering two genes *in vivo*, the growth of the breast cancer lesion was significantly inhibited, indicating that the fusion of the two mechanisms induced strong apoptosis, indicating that the dinuclear acid model of a single carrier could enhance the anti-cancer ability of TRAIL.

#### 3.1.3 Polyamide amine (PAMAM)

The non-viral vector PAMAM dendrimer is a cationic polymer carrier with gene transfer capability (Mekuria et al., 2021). Compared with traditional simple linear polymers, dendrimers also have better physical and chemical properties than conventional simple linear polymers (Daneshvar et al., 2013). PAMAM dendrimers have several unique functions that make them ideal for gene transfer, including good controllability and proportionally adequate molecular size and weight, non-immunogenic, three-dimensional branched chain structure, positively charged surface, easy surface modification, etc. (Chen et al., 2022). Cancer monotherapy might not achieve good lesion control due to incomplete regression and metastasis of the tumor. Combined therapy allows multiple drug delivery and has synergistic anti-cancer effects. Based on these, Zhang et al. (Zhang N. et al., 2022) developed a nano delivery system based on dendrimers (PAMAM) and pH-responsive liposomes (pRL) to co-deliver fluoroquinoline (FRU) and DOX for anti-vascular therapy and chemotherapy. The particle size of the nanosystem was 120 nm, and the zeta potential was 15 mV. After targeting tumor cells, FRU and DOX were released in the acidic environment of tumor cells and effectively accumulated at the tumor site to enhance tumor inhibitory effects.

The efflux of hydrophobic chemotherapeutic drugs is one of the common mechanisms of MDR, in which P-glycoprotein (P-gp)-mediated MDR is the most prominent. P-gp is a protein encoded by the MDR1 gene, which can protect normal tissues from exogenous substances. Although small drug molecules can block P-gp, P-gp inhibitors are often associated with serious adverse reactions and should be inhibited at the gene level (Waghray and Zhang, 2018; Zhang et al., 2021b). Yalamarty et al. (Yalamarty et al., 2022) prepared an amphoteric triblock copolymer based on PAMAM dendrimer for simultaneous delivery of DOX and MDR1 siRNA. The micellar system modified with 2C5 was shown to reduce toxicity and target tumor cells. The experimental results showed that the delivery carrier had excellent stability, outstanding biocompatibility and was hemolytic. The formulation could protect siRNA from degradation, which could be delivered to the target site and achieve lysosome escape. However, there was no research progress on the delivery of dual genes based on PAMAM until Li et al. (Li J. et al., 2018) designed a PAMAM-mediated co-delivery system for siRNA and pDNA for EGFR-targeted tumor therapy. In the future, we expect relevant research institutions to optimize the formula, make a carrier model suitable for delivery, and conduct *in vivo* experiments to verify the effects.

### 3.2 Natural polymer-based nanoparticles

Natural polymer-based nanoparticles have also been widely investigated in dual drug delivery systems (Bharadwaz and Jayasuriya, 2020). Due to their positively charged surface, some natural polymers are great carriers to deliver drugs and genes simultaneously. Therefore, biopolymers are excellent alternatives to carry chemotherapeutic agents and nucleic acids, such as liposoluble drugs and pDNA (Idrees et al., 2020).

#### 3.2.1 Poly (l-lysine) (PLL)

Cationic polymers are the research focus of gene delivery vectors because of their excellent gene compression ability, controllable molecular weight and easy modification. PLL are cationic polymers employed for gene transfer. Under physiological conditions, the amino acid of PLL is positively charged and interacts with negatively charged genes to form nanoparticles. Moreover, PLL has excellent biodegradability (Shi et al., 2015; Zheng et al., 2021). However, PLL is prone to aggregation and precipitation, and its poor gene transfection ability is exhibited when the ionic strength changes or is not modified (Sun et al., 2012). Glucose oxidase (GOx) can oxidize glucose into gluconic acid and H_2_O_2_. H_2_O_2_ can cause cancer cell death at high concentrations. Based on this principle, Du et al. (Du et al., 2019) prepared a delivery carrier to co-load Gox and paclitaxel (PTX) and used PLL to connect to mesoporous silica (MSN) through covalent coupling to investigate their anti-cancer effects. Their results showed that the nanoparticles could effectively decompose glucose, produce H_2_O_2_, achieve anti-tumor effects and enhance chemotherapeutic effects, further intensifying apoptosis.

Zhang et al. (Zhang et al., 2016) developed a triblock polymer micelle (NSC-PLL–PA) based on N-succinyl chitosan, PLL and palmitic acid. NSC was found to improve the cycle time of micelles and prevent premature metabolism. PLL is a cationic skeleton that can negatively adsorb charged siRNA. PA is a hydrophobic core used to encapsulate DOX. The average particle size of triblock polymer micelles is about 170 nm, with a zeta potential of 6.8 mV. The complex was unstable in a pH 5.3 environment, indicating that the complex could be rapidly released in the tumor microenvironment. In addition, the complex downregulated the expression of P-gp and increased the concentration of DOX in cells. This study proved the effectiveness of the micelles in reversing MDR, which could be a promising strategy for synergistic tumor therapy.

The use of siRNA has several advantages. On the one hand, siRNA delivery avoids issues associated with the nuclear membrane barrier because it works in the cytoplasm. On the other hand, siRNA can be pre-designed (Dong et al., 2019). When using cationic polymers to condense siRNA, the complexes usually form larger particles, which can be avoided using pDNA for condensation under the same conditions. In addition to the particle size problem, siRNA and vector are also prone to premature separation, resulting in low transfection efficiency (Hu et al., 2020). To obtain a stable siRNA delivery system, Kang et al. (Chang Kang and Bae, 2011) selected PLL as the delivery carrier, oligosulfonamide (OSA) as the surface modification and pDNA and siRNA as long-chain anions and short-chain anions, respectively, to prepare the siRNA/pDNA delivery carrier PLL/siRNA pDNA OSA complex. The results showed that the particle size of the complex was 200 nm, and the PLL/siRNA pGFP complex containing OSA had a better ability to induce gene silencing than its counterpart without OSA.

#### 3.2.2 Hyaluronic acid (HA)

Hyaluronic acid is a natural polysaccharide composed of 2000–25,000 disaccharides. The basic sugar units are glucuronic acid and acetylglucosamine, and different molecular weights can be selected according to needs [67]. Because hyaluronic acid contains carboxyl groups, it can be almost completely ionized under normal physiological pH conditions, thus showing negative charge characteristics (anionic characteristics) at indicated pH. The chemical structure of hyaluronic acid also contains other chemical groups, such as carboxyl, hydroxy and acetamido, which can be used to modify the structure with other materials. In the field of drug delivery systems, the interaction of HA-CD44 receptors has been widely used as an active tumor-targeting strategy. CD44 receptor-mediated endocytosis in cancer cells and the tumor-targeting ability of CD44 receptor-mediated hyaluronic acid-based nanosystems *in vivo* have been widely identified in various cancer cells, such as breast cancer, glioblastoma, liver cancer, lung adenocarcinoma and melanoma (Misra et al., 2015; Thapa and Wilson, 2016; Chaudhry et al., 2021).

Prostate cancer is the second largest cancer in males after lung cancer, and it is easy to metastasize (Misra et al., 2015). Inhibiting androgen secretion is one of the methods to treat metastatic prostate cancer, but it usually requires medical castration, causing pain to patients. DTX combined with steroids is the first choice for chemotherapy. Formonetin (FMN) is an effective ingredient extracted from Trifolium pratense and Astragalus membranaceus, which can be used to treat castration-tolerant prostate cancer (Tay et al., 2019). To improve the synergistic anti-cancer effects, Dong et al. (Dong et al., 2022) used HA and GE as the carrier of FMN and DTX, respectively, and self-assembled the two carriers to prepare binary nanoparticles (HA/GE-DTX/FMN NP). The results showed that the particle size of HA/GE-DTX/FMN nanoparticles was 190 nm, and the zeta potential was 20 mV. The cell uptake efficiency of HA/GE-DTX/FMN NPs was 59.6%, which had significant growth inhibition on PC3 cells. In addition, the anti-tumor efficiency of the nanocomposite was 2 times higher than the control group and free drugs and demonstrated promising synergistic anti-tumor effects.

Since breast cancer can metastasize to the lungs, liver and other organs, the 5-year survival rate of breast cancer patients is very low. The signal transducer and activator of transcription 3 (STAT3) is one of the important proteins in cells that can transmit extracellular signals to the nucleus (Yu et al., 2014). Abnormal expressions of STAT3 are often detected in breast cancer patients. It was reported that the inhibition of STAT3 could reduce tumor growth and metastasis. Therefore, STAT3 has become an attractive therapeutic target for cancer metastasis (Lee et al., 2019). Luo et al. (Luo et al., 2021) first grafted polyethyleneimine onto poly (L-lactic acid)-lipoic acid to form cationic micelles with PTX. The final delivery system was formed by combining siSTAT3 with electrostatic interaction and wrapping the cationic complex with HA. The HA coating was shown to reverse the electrical properties of the complex and increase cell uptake efficiency and targeting. The particle size of the micelle was 200 nm and its zeta potential was −21 mV. It was also proved that PTX and siSTAT3 had synergistic effects and could increase the apoptosis of tumor cells.

#### 3.2.3 Chitosan (CH)

Chitosan is a natural macromolecular nanomaterial (Lee et al., 2019). Due to its wide source, lack of immunogenicity, easy modifiability and good safety, biocompatibility and biodegradability with free amino groups, it has become one of the most modern drug carriers (Matalqah et al., 2020). However, chitosan is only soluble in acidic solutions and insoluble in neutral and alkaline solutions, resulting in poor stability of nanoparticles, which greatly limits the embedding efficiency of some drugs. Therefore, to ensure that chitosan nanomaterials can be safely used in practice, it is necessary to structurally modify chitosan in clinics, such as constructing water-soluble chitosan derivatives, amphiphilic chitosan derivatives, *etc* (Wang W. et al., 2020). Niloofar et al. (Ghobadi-Oghaz et al., 2022) synthesized a nano polymer with zein as the core and chitosan as the shell for the co-delivery of Cur and berberine (Ber) to treat breast cancer. The particle size of the nano polymer (Cur-Z-Ber-Ch) was 168 nm, the zeta potential was 36 mV, and the encapsulation efficiency of the composite to Cur and Ber was 75% and 60%, respectively. *In vitro* studies showed that the Cur-Z-Ber-Ch nanoparticles could increase cell uptake and apoptosis and significantly inhibit IL-8 proinflammatory cytokines.

Although naturally and positively charged chitosan can compress negatively charged siRNA through electrostatic interaction, its application in siRNA delivery is limited by low transfection efficiency due to low charge density and lysosomal escape efficiency (Ragelle et al., 2013). Cation modification, such as quaternization or low molecular polycation, can improve the transfection performance of chitosan (Mao et al., 2010). Studies have shown that amphiphilic drug carriers can not only encapsulate siRNA and chemotherapy drugs together but also release the two drugs to their respective targets. Yin et al. (Yin et al., 2020) developed an amphiphilic drug delivery carrier based on chitosan to co-deliver hydrophilic siRNA and hydrophobic chemotherapy drugs (PTX). HA coating was added to the delivery carrier and demonstrated good *in vivo* stability and high drug loading. In addition, disulfide bond cleavage under high GSH concentration enhanced siRNA transfection, improved the function of PTX, and made the two agents achieve synergistic effects. Because cancer is usually associated with overexpression of growth factors, it has become a research hotspot to inhibit the expression of related genes through siRNA. To solve the limitation of insufficient single gene silencing therapy, Lee et al. (Lee et al., 2015) developed a novel dual siRNA targeted delivery system for the targeted gene therapy of vascular endothelial growth factor (VEGF) and B cell lymphoma 2 (Bcl-2). The two siRNAs were chemically modified and mixed to be adsorbed by ethylene glycol chitosan nanoparticles to obtain the final carrier. Their results showed that the dual siRNA delivery system uniformly delivered each siRNA to a single cell, showing ideal siRNA therapeutic *in vivo* and great advantages in silencing the two genes.

## 4 Inorganic-based nanocarriers for co-delivery

**FIGURE 4:**
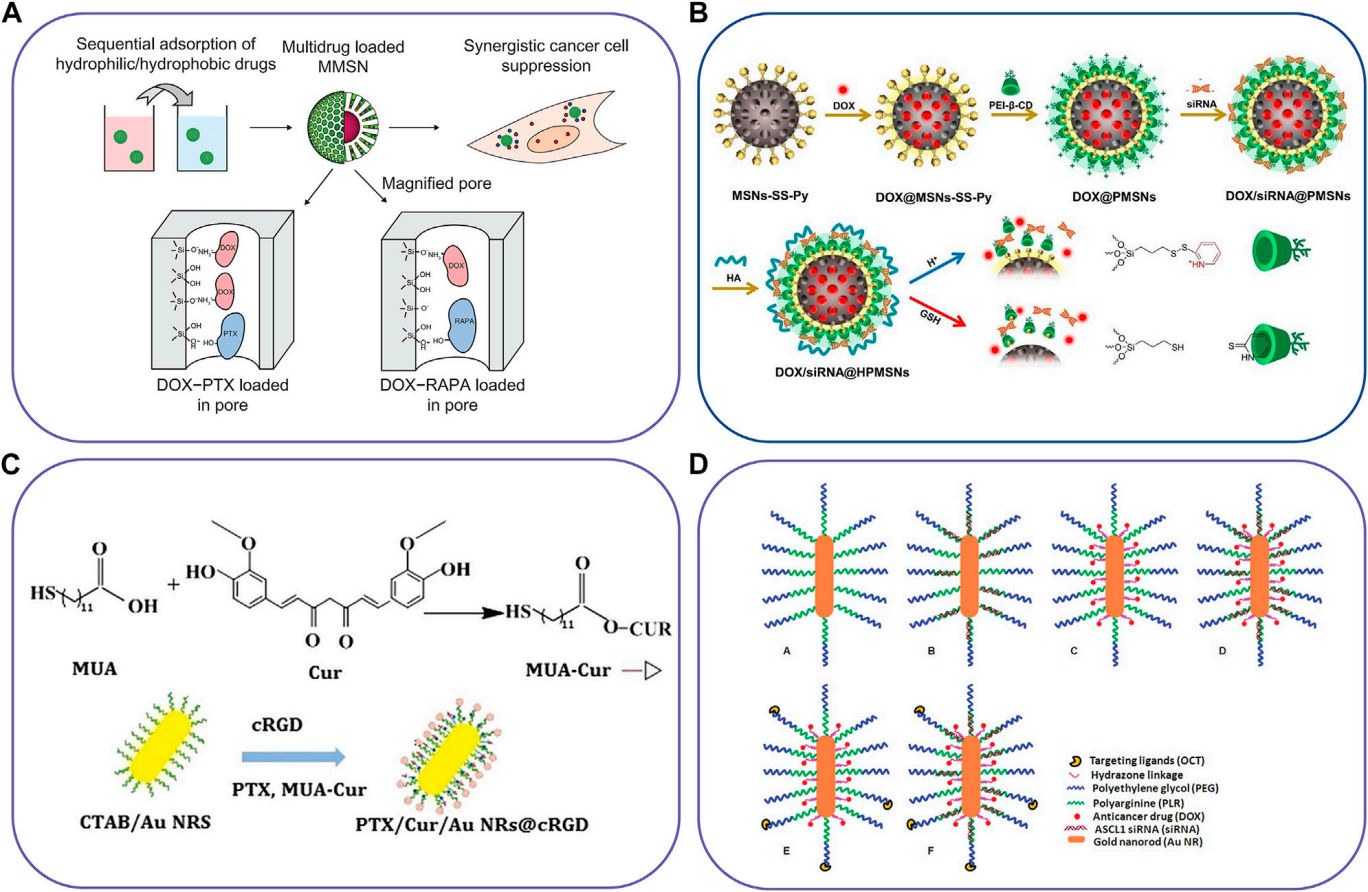
Schematic illustration of inorganic-based nanocarriers to co-deliver two different agents. **(A)** The magnetic mesoporous silica nanoparticles for co-deliver of DTX and PTX/RAPA, **(B)** pH/redox dual-responsive nanosystem to delivery DOX and siRNA, **(C)** Gold nanorod vesicles for co-delivery of CUR and PTX, and **(D)** Octreotide-conjugated gold nanorods for co-delivery of DOX and siRNA. Reproduced with permission from ref. (Liu et al., 2012), (Yuan et al., 2021), (Zhu et al., 2019), (Xiao et al., 2012). Copyright 2012, 2021, 2019 and 2012, Dovepress, Elsevier, BioMed Central, and Royal Society of Chemistry, respectively.

Inorganic nanocarriers are widely used in biomedicine due to their chemical and physical stability, tolerance to most organic solvents, low toxic side effects, controllable laboratory production cost and large surface area (Mai and Meng, 2013). Among them, the large surface area provides high drug loading and precise controlled release for drug delivery carriers. Inorganic nanoparticles often need surface modification by combining with polymers and lipids to achieve successful drug delivery (Vivero-Escoto et al., 2010). Although inorganic nanoparticles have received extensive attention in many fields, such as cell labeling and cell separation, only a few drug delivery carriers have been approved until now, and the co-administration of inorganic nanoparticles has not been approved for marketing. Currently, many inorganic nanoparticles have been studied in the laboratory, such as mesoporous silica nanoparticles, gold nanoparticles, quantum dots and iron-based nanoparticles (Niemeyer, 2003). Some novel examples about inorganic-based nanocarriers to co-delivery two different agents have shown in Figure 4 and Table 3.

**TABLE 3:** Inorganic-based nanocarriers for simultaneous delivery of therapeutics for cancer treatment.

Nanocarrier type	Nanocarrier composition	Therapeutics	Cell lines	Indication	References
MSNs	HHG_2_C_18_-L, MSN-NH_2_	Erlotinib/doxorubicin	A549	Lung cancer	He et al. (2016)
Aginate/CaCO_3_	Sodium alginate, anhydrous calcium chloride, anhydrous sodium carbonate	Doxorubicin/paclitaxel	Hela	Cervical cancer	Wu et al. (2014)
Fe/Au NP	FeCl_2_·4H_2_O, HAuCl_4_, FeCl_3_·6H_2_O	6- mercaptopurine/doxorubicin	MCF-7	Breast cancer	Ghorbani et al. (2018)
MSNs	CTAC, TEOS	Gemcitabine/paclitaxel	PANC-1	Pancreatic cancer	Meng et al. (2015)
MSNs	APTES, CTAB, TEOS	Doxorubicin/pDNA	QGY-7703	Liver cancer	Li et al. (2017)
SPIONPs	FeCl_3_, Na_2_SO_3_, NH_3_·H_2_O	Doxorubicin/siRNA	U251	Glioblastoma	Wang et al. (2020b)
CaCO_3_	DOTAP, mPEG-DSPE, CaCl_2_, Na_2_CO_3_	Doxorubicin/miRNA	HepG2	Liver cancer	Zhao et al. (2017)
LDHs	MgCl_2_·6H_2_O, NaOH, AlCl_3_·6H_2_O	5-fluorouracil/siRNA	MCF-7	Breast cancer	Li et al. (2014)
Au NP	HAuCl_4_·3H_2_O, SA, PVP	Imatinib/siRNA	B16F10	Melanoma	Labala et al. (2017)
Au NP	NaBH_4_, HAuCl_4_·3_2_O, PIC	Doxorubicin/sRNA	SK-OV-3	Ovarian cancer	Kotcherlakota et al. (2017)

### 4.1 Inorganic-based nanocarriers for delivery of drugs and drugs

Inorganic nanocarriers usually comprise an inorganic core and surface coating (Zhang et al., 2021a). The inorganic core has stable physicochemical properties, may be accurately and precisely prepared, and is the rigid support of drug carriers (Ponton et al., 2020). The surface coating can be modified to improve the functionality of inorganic delivery carriers and achieve anti-tumor treatment.

#### 4.1.1 Mesoporous silica nanoparticles (MSNs)

Owing to its large surface area, high porosity, good biocompatibility and easy surface modification, mesoporous silica is helpful for drug encapsulation and improving drug delivery efficiency (Lin et al., 2021). To achieve the synergistic effect of topoisomerase inhibitors, Li et al. (Li et al., 2013) prepared a stimulus-response vector CPT@MSN-hyd-DOX by co-delivering DOX and camptothecin (CPT), two drugs without overlapping toxicities and cross-resistance, for the treatment of glioblastoma. DOX was connected to the carrier by a hydrazone bond, and CPT was dispersed in the space of the mesoporous silica carrier. Through the rapid release of DOX under acidic conditions, CPT was released in a free diffusion manner to synergize the effects of DOX and CPT and maintain the relative “stealth” characteristics in the circulation process.

It is well known that liposomes have hydrophilic and hydrophobic properties and can deliver hydrophilic and hydrophobic drugs together. The use of inorganic nanocarriers in delivering multiple drugs can be challenging due to their uniform and stable structures; however, it was found that mesoporous silica as a delivery carrier could solve this issue (Wang Y. et al., 2015). Advanced magnetic mesoporous silica nanoparticles were developed by Liu et al. to deliver hydrophilic–hydrophobic anti-cancer drug pairs (Liu et al., 2012). Two drug combinations, DOX-PTX and DOX-rapamycin (RAPA), were prepared through sequential adsorption. The experimental results showed that the aperture of MMSN was approximately 50 nm and had a high drug-loading capacity. Compared to single-drug loaded MMSNs, multidrug-loaded MMSNs could be endocytosed by A549 cells, leading to growth inhibition effects. One strategy to increase drug loading was to design coatings on the surface of inorganic-based nanoparticles, which might help avoid drug leakage. To overcome these challenges and achieve drug synergy, Luo et al. (Luo et al., 2022) developed a mesoporous silica nanoparticle system with lipid bilayer (LB) coating to be co-administered with 3M-052 (TLR7/8 agonist) and irinotecan for the treatment of pancreatic ductal adenocarcinoma. The C18 lipid tail of 3M-052 was attached to the coated LB, and irinotecan was dispersed in pores. The combination formula not only improved pharmacokinetics but also enhanced the immunogenic cell death induced by irinotecan. In pancreatic carcinoma models, the advanced co-delivery systems led to tumor shrinkage with reduced regulatory T cells and enhanced CD8^+^ T cells.

Supramolecular photosensitizers (supraPSs) have emerged as effective photodynamic therapy (PDT) agents. By coating tirapazamine (TPZ)-loaded mesoporous silica nanoparticles (MSNs) with layer-by-layer assembled multilayer, the versatile nanoplatform (TPZ@MCMSN-Gd^3+^) was obtained with the formation of supraPSs *via* host-guest interaction and the chelation with paramagnetic Gd^3+^ (Chen W. et al., 2017). The delivery systems could be specifically uptaken by CD44 receptor and trigger the release of therapeutics. As confirmed by *in vivo* studies, the complexes showed preferential accumulation in tumor site and significantly inhibited the tumor progression by the collaboration of PDT and bioreductive chemotherapy under NIR fluorescence/MR imaging guidance.

#### 4.1.2 Magnetic nanoparticles

Magnetic nanoparticles have been used in many medical fields, such as targeted drug delivery, magnetic resonance imaging, *etc* (Vangijzegem et al., 2019). When the external magnetic field is applied, the magnetic nanoparticles gain magnetism but lose magnetism when the external magnetic field is removed (Li et al., 2021). Therefore, this feature can be explored to deliver chemotherapy drugs to the required specific location by applying an external magnetic field to play a targeted delivery function. In this context, Chiang et al. (Chiang et al., 2014) prepared pH-sensitive double emulsion nanocapsules containing trastuzumab by combining magnetic nanoparticles with single-component polyvinyl alcohol (PVA) through an emulsion process to co-deliver hydrophilic DOX and hydrophobic PTX. The results showed that the release of PTX and DOX increased when the carrier was in an acidic pH environment. Confocal microscopic images also verified the targeted binding ability of trastuzumab. Under this condition, the external magnetic field was applied to increase the HER-2 cell inhibition rate, which indicated that the magnetic targeted drug delivery system played an excellent role in cancer therapy.

Another remarkable application of magnetic nanoparticles was created by Fang et al. (Fang et al., 2014), who utilized polyacrylic acid (PAA) and iron oxide (IO) to prepare Lactoferrin (Lf)-tethered magnetic double emulsion nanocapsules. The nanoparticles could co-deliver DOX and CUR encapsulated in the core and shell, respectively. Drug targeting was achieved by applying an external magnetic field, while drug release was achieved by regulating the surface charge. These core-shell W/O/W magnetic nanocapsules were effectively delivered into RG2 glioma cells. Through the combination of magnetic application and Lf ligand, the agents’ cell uptake was significantly increased in RG2 cells, resulting in drug accumulation and inhibiting tumor growth effectively. The combination of passive and active targeting is also one of the research emphases of magnetic nanoparticles. Hiremath et al. (Hiremath et al., 2019) prepared Fe_3_O_4_ nanoparticles with an oleic acid shell using an alkaline co-precipitation method for the co-delivery of DOX and CUR. The size of the nanocomposite carrier was 12.5 nm, and the drug encapsulation rates of compactaxel and CUR were 43.7% and 56.5%, respectively. The targeting efficiency and cellular uptake were eventually increased under the action of an external magnetic field.

#### 4.1.3 Other inorganic -based carriers

Stimulation response nanocarriers have received increasing attention in recent years (Fernandez and Orozco, 2021). When there are specific chemical bonds in the delivery system, the corresponding response can be applied to break the chemical bonds to achieve the purpose of drug release. Lighting is an external stimulus and owing to its low cost and minimally invasive to the organization, near-infrared light has been used in drug delivery systems (Yu et al., 2021). In addition, near-infrared light can use light absorbers, such as gold nanoparticles, to promote photothermal therapy for cancer treatment. Zhu et al. (Zhu et al., 2019) prepared a cRGD peptide-modified gold nanorod to co-deliver PTX and CUR for chemophotothermal cancer therapy. The banding of cRGD to αvβ3 promoted the endocytosis of nanocarriers, enhanced the response of gold nanorods, improved near-infrared enhanced drug release and strengthened therapeutic efficiency. This innovative therapy provided a versatile strategy for the precise treatment of tumors.

As the powerful redox potential and phototherapy, researchers started to use copper as a drug delivery carrier (Naikoo et al., 2021). Copper nanoparticles can alter gene regulation and cell shape and increase the apoptosis of cancer cells through DNA destruction. Guo et al. (Chen et al., 2018) co-delivered DOX and DTX into CuO nanoparticles for the targeted delivery therapy of nasopharyngeal carcinoma. A PLGA external shell was covered on the surface of CuO for connecting folic acid. The research results showed that the external coating enabled the CuO nanoparticles to have an extremely high drug loading rate and significant stability, enhanced the drug circulation and release, and exhibited considerable efficacies in destroying prostate cancer cells (DU145-TXR). Nanographene oxide (NGO) has been widely studied for drug delivery due to its large surface area, easy conductivity and high drug-loading efficiency. In addition, there are hydrophilic groups such as hydroxyl and carboxyl groups on both sides of the NGO surface, which can increase the stability of the carrier in the solution. Yang et al. (Yang et al., 2013) developed an advanced NGO drug delivery nanoparticle co-loaded epirubicin (EPI) and anti-EGFR antibody (C255) with PEG connection for blocking EGFR growth signal to treat glioma. The experiment on glioma cells (U87) showed that PEG-NGO-C225 caused a significant decrease in EGFR expression, prolonging the survival of U87 tumor-bearing mice for 50 days. NGO-based nanoparticles may allow toxic drugs to be safely delivered to tumor cells to overcome the challenges of tumor treatment.

### 4.2 Inorganic-based nanocarriers for delivery of drugs and genes

Compared to organic-based gene delivery systems, inorganic-based gene delivery systems are still in the development stage. Some specific properties of inorganic nanoparticles, including magnetism and biological stability, provide unique delivery opportunities for these systems. However, many shortcomings such as high price and complicated treatment steps still limit their wide application.

#### 4.2.1 Gold nanoparticles

Au NPs have shown considerable potential in the field of drug delivery and are considered a pharmaceutical breakthrough (Singh et al., 2018). In addition, the inner core of Au NPs is inert and non-toxic, and the modification of chemical bonds can impart more functions (Fan et al., 2020). Moreover, the improved gold nanoparticles have biocompatibility and non-immunogenicity and can be used as radiosensitizers and photothermal agents in the medical field. Li et al. (Li et al., 2015) developed modified gold nanoparticles to co-deliver captopril and siRNA for the treatment of breast cancer. Captopril was connected with PEI through an amide bond, and the remaining thiol group was connected with polyethylene imine (CP). Finally, siRNA was loaded by electrostatic adsorption to form a siRNA/CP/GNP complex. The particle size of the complex was approximately 87 nm, demonstrating effective EPR effects and gene-silencing properties. Captopril and siRNA are delivered to the same cell together, which leads to significant downregulation of VEGF by Ang II-ACE and siRNA-dependent pathways in MDA-MB435 cells, resulting in stronger inhibition of angiogenesis.

To enhance the therapeutic outcomes of neuroendocrine (NE) cancers, Xiao et al. (Xiao et al., 2012) developed a nanocarrier based on Au NR, which could co-deliver DOX against ASCL1 siRNA using octreotide (OCT) as the active solvent targeting ligand to target NE cancer cells overexpressing SSTRs. To achieve drug release controlled by pH-sensitive, DOX was connected with the Au NRs *via* specific chemical bonds, and siRNA was conjugated onto the Au NRs by electrostatic interaction. The results showed that the gene silencing efficiency of Au–DOX–OCT–ASCL1 siRNA systems in BON carcinoid cancer cells was significantly higher than that of Au–DOX–siRNA nanoparticles and exhibited an excellent therapeutic efficiency in treating NE cancer cell lines. In a study performed by Yin et al. (Yin F. et al., 2015), gold nanorods-based nanocarriers were employed to conjugate DOX and mutant K-Ras siRNA to treat pancreatic carcinoma. With 665 nm light reflection, DOX and siRNA were released simultaneously to the Panc-1 cells to achieve a synergistic effect and effectively inhibit tumor growth. Such an approach was shown to downregulate K-Ras gene expression and exert powerful anti-proliferative effects against pancreatic cancer cells.

#### 4.2.2 Mesoporous silica nanoparticles (MSNs)

Mesoporous silica nanoparticles (MSNs) are major inorganic-based nanoparticles applied in co-delivery (Zhou et al., 2018). The high surface area of MSNs makes them adequate to be extensively used in chemotherapy (Castillo et al., 2020). Moreover, the coating of their chemical shell makes it easier for them to encapsulate agents. To regulate P-gp levels in drug-resistant cancer cells, Yuan et al. (Yuan et al., 2021) fabricated a nanosystem (HPMSN) to co-deliver DOX and GCN5 siRNA with a hyaluronic acid-coated to trigger pH/redox dual-responsive. This nano delivery system effectively released DOX and siRNA *via* pH/redox dual responsiveness and restricted leakage during the initial circulation. With the assistance of HA shell, HPMSN enhanced drug internalization through CD44-mediated targeting. In breast tumor model (MCF7/ADR) experiments, GCN5 siRNA downregulated P-gp levels and eliminated P-gp-mediated drug resistance. The results showed that HPMSNs inhibited tumor growth by 78% and minimized systemic toxicity of DOX.

Another example of an external shell is the cross-linked polyethylene imine-coated drug/siRNA co-delivery carrier based on mesoporous silica by Zhang et al. (Zhang R. et al., 2022), which demonstrated good diagnostic sensitivity and drug delivery. There are numerous pores on the surface of fluorescent mesoporous core-shell silica. Polyethyleneimine is cross-linked on the negatively charged surface by electrostatic interaction and combines with negatively charged siRNA. The disulfide bond reduces the carrier’s response, allowing the release of drugs according to different GSH concentrations. Therefore, fluorescent silicon nuclei can be used as fluorescent probes to track the cellular uptake of carriers. Babaei et al. (Babaei et al., 2020) developed mesoporous silica nanoparticles for co-delivery of camptothecin (CPT) and survivin shRNA to treat colon adenocarcinoma. The prepared system was rod-shaped, with a 150 nm diameter. CPT was first loaded into the MS nanoparticles, and the system was PEGylated to condense iSur-pDNA. Then, an AS1411 DNA aptamer allowed the system to provide selective therapies. The results showed that the CPT was controlled released through the system and induced a synergistic effect with iSur-pDNA in cytotoxicity. In addition, the AS1411 DNA aptamer increased drug uptake in cancer cells resulting in cellular toxicity and apoptosis.

#### 4.2.3 Other inorganic-based carriers

Inorganic anions such as CO_3_^2-^ can promote the co-precipitation of Ca^2+^ with DNA and have unique advantages in gene delivery systems (Dizaj et al., 2015). To improve drug delivery efficiencies, Zhao et al. (Zhao et al., 2012) fabricated calcium carbonate-based nanoparticles. A cell-penetrating peptide, KALA, was introduced into this system and luciferase reporter gene plasmid was coated on the system’s surface. Due to the existence of KALA, the cellular uptake and gene expressions caused by CaCO_3_-KALA-DNA were significantly enhanced in 293T cells. Moreover, p53 expression plasmid and DOX were encapsulated in CaCO_3_-KALA-DNA to verify the co-delivery efficiency. The results showed that CaCO_3_-KALA-p53-DOX nanoparticles exhibited strong delivery efficiency and could significantly inhibit HeLa cells. Due to the tunable properties of molecular building blocks, nanoscale metal-organic frameworks (MOF) can serve as efficient nanocarriers for drug delivery. Similar to mesoporous silica, MOF also has the property of high porosity and can be surface modified as well. Chemotherapeutics can be loaded in large pores, and siRNA can be linked to metal ions on the MOF surfaces. He et al. (He et al., 2014) co-loaded a cisplatin prodrug and siRNA into NMOF to block MDR pathways. Cisplatin and siRNA can be simultaneously delivered to ovarian cancer cells, and siRNA was protected from nuclease degradation by UiO to enhance cellular uptake. As a result, the NMOF could be a promising strategy for treating ovarian cancer.

Carbonate apatite has drawn increasing as a promising inorganic component for drug delivery systems (Takahashi et al., 2018). It can be synthesized by calcium phosphate precipitation through bicarbonate. Its particle size can be controlled between 50–300 nm by crystal growth dynamic (Uddin et al., 2018). These carriers dissolute fast in endosomal acidic pH, thus causing an effective release of therapeutic drugs. To change the constraints of pharmacokinetics, Fatemian et al. (Fatemian et al., 2019) developed inorganic carbonate apatite (CA) nanoparticles to co-encapsulate against AKT/ERBB2 siRNA and paclitaxel (PTX) for breast cancer treatment. Compared with the CA-PTX, CA-PTX-siRNA had the largest anti-cancer effects in 4T1 cells. *In vivo* investigations showed that the group of CA-PTX-siRNA had smaller tumors than the CA-PTX group. The carrier might be a potential approach for improving the survival of cancer patients.

### 4.3 Inorganic-based nanocarriers for delivery of genes and genes

#### 4.3.1 Mesoporous silica nanoparticles (MSNs)

MSNs have attracted great attention as potential gene delivery systems due to their good stability and easy-to-modify high surface area. Wang et al. (Wang Y. et al., 2020) developed iRGD-modified mesoporous silica nanoparticles for the co-delivery of siPlk1-siRNA and miR-200c-miRNA for metastatic cancer treatment. Indocyanine green (ICG) coating on the carrier surface can promote the connecting ability of iRGD to achieve targeted binding. The results showed that ICG could promote lysosomal escape and RNA release, kill metastatic breast cancer cells and inhibit tumor growth. As another example of using mesoporous silica (MS), Shahidi et al. (Shahidi et al., 2022) co-delivered miR-34-miRNA and siPD-L1-siRNA to treat bladder cancer. These c (RGDfK)-modified MS nanoparticles showed good blood stability and effective RNA release. In addition, this nanocarrier demonstrated biological stability in the serum environment and effectively protected RNAs against degradation. Upon releasing RNAs, adjustments of PD-L1 and miR-34a resulted in a decreased expression of CD44 and inhibited the growth of cancer cells in bladder tumor models.

#### 4.3.2 Other inorganic-based carriers

Nucleic acids decompose rapidly in plasma and are negatively charged under physiological conditions, which limits their entry into the cells. A specific delivery system is needed to ensure the delivery of nucleic acid drugs to the target tissue. Cationic gold nanoparticles can be combined with negatively charged nucleic acids and delivered to target cells. Some studies showed that the surface modification of AuNP can improve its stability, such as in cationic carbosilane dendrites (Alle et al., 2022). It is possible to realize polygenic delivery of nanoparticles through LbL assembly technology. Bishop et al. (Bishop et al., 2015) used gold nanoparticles as the core and degradable polymers as the coating, which were added layer by layer to the surface of gold nanoparticles to deliver DNA and siRNA. When each layer of polymer is coated, the zeta potential will be reversed to achieve the purpose of drug loading. The final composite nanoparticles have a particle size of about 200 nm and can be internalized by human primary brain cancer cells. It was observed by TEM that better gene knockout efficiency and foreign DNA expression could be obtained in cells. Thus, it can be seen that LbL assembly technology may provide gene combination therapy for cancer treatment.

Carbonate apatite (CO_3_Ap) nanoparticles have also been widely studied as drug delivery systems due to efficient endocytosis and rapid response to pH *in vivo* (Alhaji et al., 2014). To overcome MDR, Li et al. (Li et al., 2012) used CO_3_Ap nanoparticles to co-deliver siRNAs targeting ABCG2 and ABCB1 gene transcripts to reverse MDR in breast cancer and improve sensitivity to chemotherapeutic drugs. The results showed that the siRNA introduced into cells enhanced the sensitivity to chemotherapy drugs and dose-dependently increased chemosensitivity. The nanocarrier has a high application prospect in clinical research, which may be applied in multi-drug resistance settings of malignant tumors in the future.

## 5 Future perspectives and conclusion

The mechanism of tumor genesis and development is complex, requiring different drugs for different targets. With the development of RNAi technology, gene-level cancer treatment is becoming a reality, and nucleic acids, such as siRNA, miRNA, shRNA, etc., are being used as drugs for cancer treatment. Despite great achievements in single-drug delivery, the mechanism of MDR is complex; thus, combination chemotherapy is extremely important, but the possibility of increased risks of adverse events and poor compliance should be carefully considered, especially when multiple drugs are being simultaneously administered. Therefore, one delivery carrier simultaneously delivering multiple drugs could significantly improve patient compliance and therapeutic effects. To achieve accurate targeted delivery, the delivery and release of drugs using nanocarriers seem a promising strategy. Due to the different physical and chemical properties of chemotherapeutic drugs, such as hydrophilicity and hydrophobicity, gene drugs and chemotherapy agents also have differences in positive and negative charges. Therefore, future research in this field should focus on developing multifunctional nanocarriers and delivery mechanisms using appropriate materials to package drugs or genes and carrying out reasonable modifications to achieve accurate delivery. Although there are many difficulties in combined delivery, some progress have made in synergistic therapy. Vyxeos (CPX-351) was the first dual drug liposome approved by FDA in 2017, and it is a liposomal nanoparticle for the treatment of acute myeloid leukemia (AML) that incorporates the drugs cytarabine and daunorubicin (Lancet et al., 2018). In addition, Tabernero et al. (Tabernero et al., 2013) initiated a trial of ALN-VSP, an LNP formulation of siRNAs targeting VEGF and KSP, in cancer patients. These data of clinical trial form the basis for further development in cancer.

In this review, we focused on the commonly used materials and dosage forms of co-delivery nanocarriers, discussed the research progress of different vectors, described the challenges and strategies in the delivery process, and highlighted future developmental prospects. Although the combined delivery still has some shortcomings, such as insufficient stability of the delivery carrier, difficult preparation process and miss target effect, *etc*., with increasing research in this field, the synergistic delivery strategy might be implemented in future cancer treatment.
